# Atypical Fibroxanthoma: An unexpected cause of hemoptysis

**DOI:** 10.15388/Amed.2021.28.1.16

**Published:** 2021-04-29

**Authors:** Konstantinos Mantzouranis, Vasiliki Epameinondas Georgakopoulou, Serafeim Chlapoutakis, Despoina Melemeni, Christos Damaskos, Nikolaos Garmpis, Pagona Sklapani, Nikolaos Trakas, Xanthi Tsiafaki

**Affiliations:** 1^st^ Pulmonology Department Sismanogleio Hospital, Athens, Greece; Pulmonology Department, Laiko General Hospital, Athens, Greece; Department of Thoracic Surgery, Agios Savvas Hospital, Athens, Greece; 1^st^ Pulmonology Department Sismanogleio Hospital, Athens, Greece; Second Department of Propedeutic Surgery, Laiko General Hospital, Medical School, National and Kapodistrian University of Athens, Athens, Greece N.S. Christeas Laboratory of Experimental Surgery and Surgical Research, Medical School, National and Kapodistrian University of Athens, Athens, Greece; Second Department of Propedeutic Surgery, Laiko General Hospital, Medical School, National and Kapodistrian University of Athens, Athens, Greece N.S. Christeas Laboratory of Experimental Surgery and Surgical Research, Medical School, National and Kapodistrian University of Athens, Athens, Greece; Department of Cytology, Mitera Hospital, Athens, Greece; Department of Biochemistry, Sismanogleio Hospital, Athens, Greece; 1^st^ Pulmonology Department Sismanogleio Hospital, Athens, Greece

**Keywords:** atypical fibroxanthoma, pulmonary metastases, hemoptysis

## Abstract

Atypical fibroxanthoma is an infrequent, low-grade superficial cutaneous neoplasm, usually presenting as a nodule or plaque of red color. It is considered as a superficial variant of pleomorphic dermal sarcoma. Although atypical fibroxanthoma has similar histologic features to pleomorphic dermal sarcoma, it has less aggressive behavior. Atypical fibroxanthoma usually occurs on sun-exposed regions of the head and neck of elderly patients. Ultraviolet light, specific genetic mutations and administration of immunosuppressive agents to transplant recipients have been associated with the pathogenesis of the tumor. The prognosis is typically excellent when treated with complete excision of the primary lesion. This report describes the rare case of a 84-year-old man with hemoptysis due to metastatic cutaneous atypical fibroxanthoma.

## Abstract (lt)

**Atipinė fibroksantoma – netikėta hemoptizės priežastis:**

**Santrauka. **Atipinė fibroksantoma yra nedažna, nedidelė paviršinė odos neoplazma, dažniausiai atrodanti kaip raudonos spalvos gumbelis ar plokštelė. Ji yra išorinis pleomorfinės odos sarkomos variantas. Nors atipinė fibroksantoma turi panašius histologinius požymius kaip ir pleomorfinė odos sarkoma, ji yra ne tokia agresyvi kaip pastaroji. Atipinė fibroksantoma paprastai atsiranda vyresniems pacientams galvos ir kaklo srityse, kurios nėra apsaugotos nuo tiesioginių saulės spindulių. Ultravioletiniai spinduliai, tam tikros genetinės mutacijos ir imunosupresantų skyrimas transplanto recipientams buvo susiję su naviko patogeneze. Prognozės dažniausiai puikios, kai visiškai pašalinama pirminė žaizda. Šiame straipsnyje aprašomas retas atvejis, kai 84 metų vyriškiui buvo diagnozuota hemoptizė, sukelta odos atipinės fibroksantomos metastazės.

**Raktažodžiai: **atipinė fibroksantoma, plaučių metastazės, hemoptizė

## Introduction

The term atypical fibroxanthoma of skin was described for the first time in 1963 in a group of cases by Helwig et al [1]. Atypical fibroxanthoma is a malignant fibrohistiocytic tumor, most frequently presented as a solitary nodule or ulceronodule on exposed skin of the head and neck in elderly white men, with rapid growth during the course of several months. Most of the tumors are asymptomatic, however ulceration or bleeding is not rare [2]. It is a diagnosis of exclusion [3]. Atypical fibroxanthoma is rare with an incidence estimated at 1.8/100.000 and has been mentioned to account for 0.002% of all nonmelanoma skin cancers [4].

Atypical fibroxanthoma represents a low-grade neoplasm of atypical spindle cells with intermediate malignant behavior. Differential diagnosis includes squamous cell carcinoma, melanoma, angiosarcoma, leiomyosarcoma and dermatofibrosarcoma protuberans [3]. Ultraviolet radiation has been reported to implicate the etiopathogenesis of the neoplasm and one study showed mutations in p53 gene in patients with atypical fibroxanthoma. Besides, atypical fibroxanthoma has developed in renal and heart transplant recipients, indicating that immunosuppression may have a role in etiopathogenesis of the disease [5].

Atypical fibroxanthoma is included in the same spectrum with pleomorphic dermal sarcoma, as both tumors are characterized by similar etiologic, histologic and clinical factors. The terminology and diagnostic criteria for the two tumors continue to be controversial. Tumors sited more superficially, with minimal subcutis involvement, can be called atypical fibroxanthoma, while pleomorphic dermal sarcoma has features like several atypical mitoses, perineural, or perivascular invasion and is associated with a poor prognosis and higher rates of metastasis [6]. Factors related to recurrence and metastasis include subcutaneous fat invasion, incomplete resection, tumor necrosis, close excision margins, poorly circumscribed tumor, lymphovascular invasion or perineural invasion [3]. Complete surgical excision is the treatment of choice for atypical fibroxanthoma [3]. The use of Mohs micrographic surgery, which allows the removal of all malignant cells while spares healthy tissue and leaves the smallest possible scar, is also supported by retrospective studies with low risk of recurrence [7]. Radiation therapy is an additional option for treatment of atypical fibroxanthoma that is traditionally reserved for adjuvant treatment of tumors that cannot be resected with clear surgical margins. In some cases, radiation therapy is given as the primary treatment for patients who are not candidates for surgical resection [3]. 

Atypical fibroxanthoma is rarely associated with distant metastases [3]. Complete surgical excision can be achieved either by wide local excision or Mohs micrographic surgery. When clear margins are achieved through these procedures, atypical fibroxanthoma has a recurrence rate of 5.6% and a rate of metastasis of 0.5%. According to all the case series that have been examined, metastatic atypical fibroxanthoma is extremely rare (0.95%) and traditionally occurs within 1–2 years after the initial diagnosis is made [8].

Herein we describe a rare case of a 84-year-old man with pulmonary metastases from cutaneous atypical fibroxanthoma presenting with hemoptysis. 

## Case Report

### Case Presentation

A 84-year-old man presented to our Pulmonology Department with cough accompanied with bloody sputum over the last month and weight loss, approximately 15 kg, over the last year. The patient had a medical history of coronary artery disease with percutaneous coronary intervention (PCI) and surgically resected head cutaneous atypical fibroxanthoma one year ago without abnormal findings at excision region within 6 months follow-up. The clinical examination was without remarkable signs.

### Investigations

**Figure 1.**
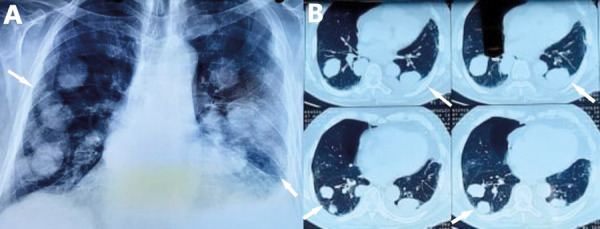
A. Chest X-ray shows pulmonary nodules in both lungs. B. Computed tomography of the chest reveals pulmonary nodules of various size and a small left pleural effusion.

Complete blood count revealed normochromic normocytic anemia and the coagulation tests were normal. The other blood biochemistry parameters were normal, with the exception of an elevated serum lactate dehydrogenase (LDH) 501 U/L (normal <225 U/L). Chest X-ray revealed multiple pulmonary nodules and left pleural effusion (Figure 1A). 

The patient underwent computed tomography (CT) of the chest and abdominal CT. Chest CT showed several nodules of various size in all lung fields, ground glass opacities and a small left pleural effusion (Figure 1B), while from abdomen CT no suspicious abnormality of solid organs was noted. 

### Differential Diagnosis

Bronchoscopy with bronchoalveolar lavage (BAL) was performed without abnormal findings. CT-guided fine needle biopsy of a lesion in the left lower lobe was conducted. Histopathological and examination of biopsy specimen demonstrated partially necrotic malignant neoplasm of spindle cells with evidence of recent and old hemorrhage. Immunohistochemical staining revealed that this neoplasm was positive for CD10, S100 and CD99. CK8/18, p63, GATA3, HMB45, CD34, podoplanin, CD68, Hepaticyte D, Desmin and PSA were negative. Morphological and immunophenotypic findings supported the diagnosis of metastatic cutaneous atypical fibroxanthoma. 

### Treatment and Follow up

The patient declined to receive any specific therapy. He died two months after the diagnosis.

## Discussion

Pulmonary metastasis from cutaneous atypical fibroxanthoma was described for the first time in 2003 by Kargi et al. [5]. Very few cases report that this neoplasm rarely metastasises to lymph nodes, parotid glands, bone marrow, skeletal muscles, brain and lungs [3,5, 9-10]. In addition, pulmonary, brain and liver metastases have been noticed in cases with minimal or no subcutaneous involvement [11]. 

Kargi et al. described a case of a 68-year-old man with was diagnosed with atypical fibroxanthoma in his right thigh and presented with respiratory symptoms within 6 months after surgical excision. Chest CT revealed pulmonary metastasis and histological examination of the lesion, that was obtained with CT-guided fine needle biopsy, demonstrated metastasis from atypical fibroxanthoma [5]. Armstrong et al. reported a case of an 82-year-old man with atypical fibroxanthoma on the scalp who presented with brain and lung metastasis secondary to this neoplasm[3].

Sheth et al. reported a case of a 41-year-old woman with atypical fibroxanthoma of her left ankle. Staging fluorodeoxyglucose positron emission tomography (FDG PET)/CT revealed local extension of the lesion to bone and metastases to lung parenchyma, lymph nodes, bone marrow and muscles [9]. Satter et al. described a case of a 63-year-old man with the diagnosis of atypical fibroxanthoma on his left temple that was completely resected with Mohs micrographic surgery. 

Thirty-one months later, the patient presented with cough, fever and imaging revealed loculated pleural effusion that did not respond to antibiotics administration and a lung biopsy demonstrated metastatic atypical fibroxanthoma to the lung [10]. 

Wang et al., in their review of 152 cases of atypical fibroxanthoma from their institution and from a consultative dermatopathology practice, reported 7 cases of atypical fibroxanthoma, with minimal or no subcutaneous involvement who presented with lung metastases [11]. Wollina et al. analyzed 25 patients with atypical fibroxanthoma from their Dermatology Department and reported only one patient with a single lung lesion suspicious for a metastasis [12]. 

## Conclusions

This is a rare case of pulmonary metastases caused by metastatic cutaneous atypical fibroxanthoma in patient presenting with hemoptysis. Clinicians should be aware of this potentially aggressive tumor and should take into consideration the possibility of recurrence and subsequent metastases. 
